# Effect of Mild Heating on Human Lens Epithelial Cells: A Possible Model of Lens Aging

**DOI:** 10.1038/srep33917

**Published:** 2016-10-11

**Authors:** Keke Zhang, Xiangjia Zhu, Yi Lu

**Affiliations:** 1 Department of Ophthalmology, Eye and ENT Hospital, Fudan University, Shanghai, China; 2 Key Laboratory of Myopia, Ministry of Health P.R. China, Shanghai, China; and Shanghai Key Laboratory of Visual Impairment and Restoration, Fudan University, Shanghai, China

## Abstract

This study aims to investigate the effect of mild heating on lens epithelial cells and to explore its possibility as an *in vitro* model for lens aging. Human lens epithelial cells (LECs) were heated at 50 °C for a cellular lens aging study. Analysis of the head group order of lens membranes was performed using Laurdan labeling. Immunofluorescence was performed to detect changes in α-crystallin expression and its cellular distribution. The chaperone-like activity of α-crystallin was also assessed. After mild heating, α-crystallin in LECs showed a tendency towards accumulation around the nucleus. The membrane head group environment of lens epithelial cells became more fluid with increasing time of exposure to mild heating, as indicated by increased water penetration. Furthermore, the chaperone activity of α-crystallin decreased, and suggests a relatively lower protective effect on other functional proteins in LECs. Thus, compared to the mild heating model based on lens tissue, this cellular model could provide a more convenient and accurate method for studying lens aging *in vitro*, including changes in membrane head group order in each cell, the real-time observation of crystallin distribution, and the monitoring of functional changes in the chaperone activity of crystallins as a result of aging.

As a cellular organ, the lens maintains its transparency owing to its complex composition of unique proteins^1^. However, the delicate balance required for lens transparency is affected by aging, thereby ultimately leading to lens opacification or even cataract. Therefore, the mechanisms of lens aging is an ongoing focus in the field of cataract research.

With aging, modifications of lens crystallins, including amorphous-aggregation and cross-linking, can contribute to protein insolubilization^2^. Previous studies^3^,^4^ showed that these major modifications are observed *in vivo* in α-crystallin^5^. As an important small heat shock protein (sHSP), α-crystallin is essential for maintaining the normal function of cellular membranes and other lens proteins. Our previous study^6^ showed that there are age-related changes in membrane head group structures in the lens. With the assistance of Laurdan labeling and microscopy experiments, Zhu *et al*. reported that the nuclear membranes of the aged lens showed a relatively decreased head group order than normal lenses^6^. Furthermore, the results from this study also support that α-crystallin, may participate in the regulation of lens membrane properties. Alpha-crystallin is also thought to prevent the amorphous-aggregation of other lens proteins that denature over time, thereby playing an important role in the maintenance of long-term lens transparency. The presence of crystallin fragments, mainly α-crystallin, has been detected in aggregates of both water-soluble high molecular weight (WS-HMW) and water-insoluble (WI) proteins in the human lens^7^,^8^. Previous analysis of aging and human age-related cataract lenses has also revealed the presence of increased levels of WS-HMW and WI proteins^9^. The amorphous-aggregation of α-crystallin and formation of WS-HMW and WI proteins may finally lead to lens opacity. Therefore, further research into changes in head group order in lens membranes and crystallin function is important in for extending existing knowledge of lens aging and cataract development.

A tissue-based mild heating model has been widely applied as an appropriate model for age-related cataract. Heating intact lenses at 50 °C has been considered a simulated condition in human lenses^10^. As the most well-known tissue to possess life-long proteins, and owing to its unique construction, the lens has been considered a favorable model for exploring the aging processes^11^. However, lens tissues are not easily accessible, especially in countries in which organ donation is still unconventional. The application of the mild heating model for aging using lens epithelial cells (LECs) would be a preferable and convenient method for the study of age-related cataract. However, to our best knowledge, no other study thus far has discussed the possibility of studying aging in the lens by utilizing a cellular model. It is known that when cells are exposed to elevated temperatures, membrane defects and permeability alternation may occur due to the induction of nonbilayer structures^1^,^6^. Accordingly, analysis of membrane head group environments and crystallin function through a cellular mild heating model could provide new insights into the controlling mechanisms behind lens aging *in vivo*.

This study aims to establish a model for lens aging *in vitro* in which changes in head group order in cellular membranes and α-crystallin function can be detected. Using the mild heating model in LECs, we have developed an innovative platform and provide a new direction for lens aging research.

## Results

### Changes in cell viability in lens epithelial cells after mild heating

**Figure 1:**
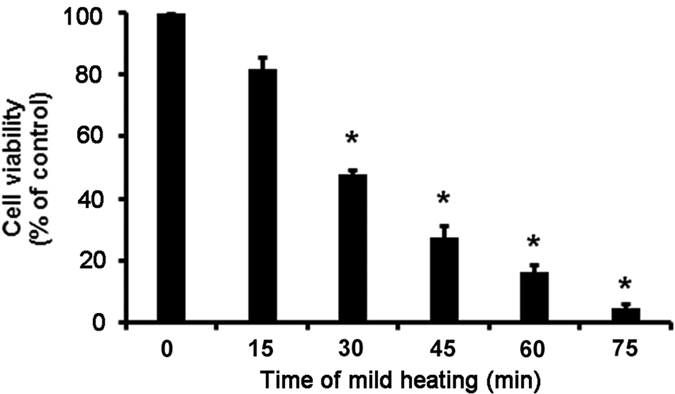
Changes in cell viability in lens epithelial cells after mild heating. Cultured human lens epithelial cells were incubated at 50 °C over different time periods. Cell viability was assessed using the CCK-8 kit. Each experiment was performed in triplicate. *P < 0.05.

Using the CCK-8 kit, we studied the viability of LECs incubated at 50 °C over different time periods (0, 15, 30, 45, 60, and 75 min). During the 75-minute mild heating incubation period, cell viability decreased with increased heating time (Fig. 1). According to our data, the majority of cells were nonviable after incubation at 50 °C for 75 min (Fig. 1). Therefore, measurements were taken at time points of 0, 15, 30, 45, 60, and 75 min during incubation, and during which cell viability showed a significant and continual decline until almost no viable cells remained (Fig. 1).

### Changes in membrane head group order in lens epithelial cells after mild heating

**Figure 2:**
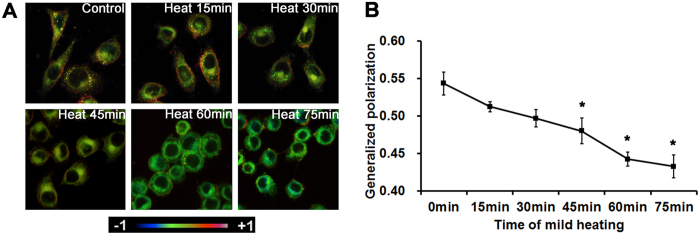
The effect of mild heating on membrane GP values of lens epithelial cells. (**A**) Representative pseudocolored GP images of lens epithelial cells indicate changes in membrane head group order following mild heating. (**B**) Membrane properties as a function of different durations of mild heating on lens epithelial cells. GP values (mean ± SD) from the cellular membrane of lens epithelial cells were calculated from the reconstructed GP images. *P < 0.05.

After mild heating, changes in the membrane head group order were observed in lens epithelial cells, as shown in Fig. 2. According to the reconstructed GP images, the GP values of cellular membranes showed a significant decrease with increasing heating time, indicating the increased fluidity of the membrane head group environment during incubation.

### Changes in intracellular distribution of α-crystallin after mild heating

**Figure 3:**
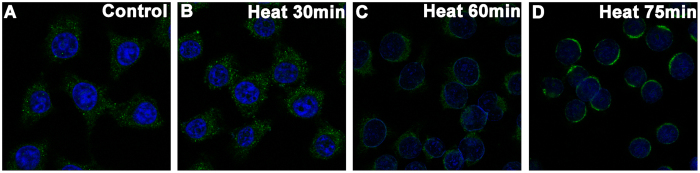
Changes in intracellular distribution of αA-crystallin during mild heating over time.

**Figure 4:**
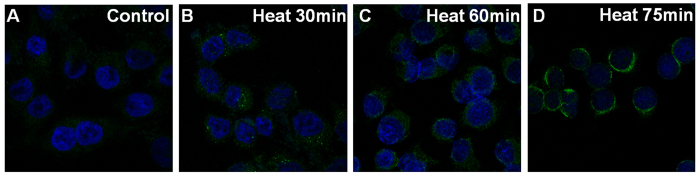
Changes in intracellular distribution of αB-crystallin during mild heating over time.

The intracellular distribution of positive signaling by αA-crystallin and αB-crystallin is shown in Figs 3 and 4. With increasing duration of heating, αA-crystallin and αB-crystallin gradually accumulated in the cytoplasm, forming a ring-like zone with high a fluorescence signal surrounding the nucleus.

### Changes in water-soluble proteins after mild heating

**Figure 5:**
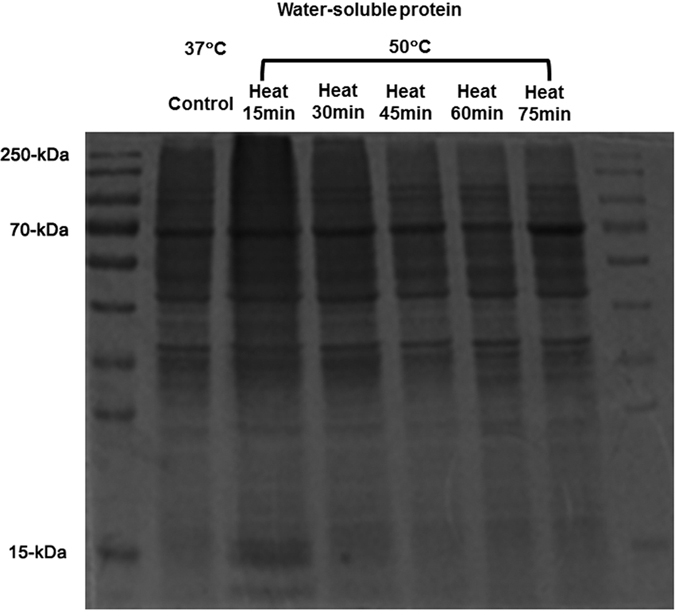
Changes in water-soluble protein expression as a result of mild heating of increasing duration.

The SDS-PAGE analysis of proteins extracted from LECs revealed two opposing trends depending on the varying molecular weight of protein bands (Fig. 5). For the WS-HMW proteins, which were represented in the 70-kDa band, there was an increase in relative expression with increasing heating time. After heating for 75 min, the 70-kDa band showed the highest expression compared to the other groups. On the other hand, the low molecular weight (LMW) proteins, represented by the 15-kDa to 30-kDa band, exhibited decreased expression with increasing heating time, and were expressed predominantly in cells which were heated for only 15 min, while showing minimal expression in cells heating for longer time periods.

### Changes in the chaperone activity of α-crystallin after mild heating

**Figure 6:**
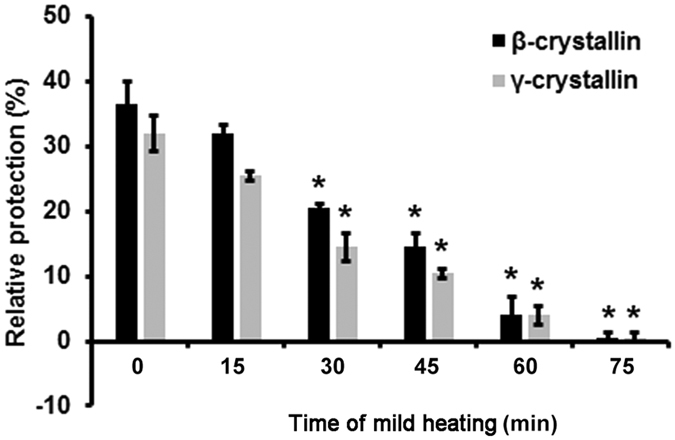
Changes in the chaperone activity of α-crystallin following mild heating. *P < 0.05.

Figure 6 shows changes in α-crystallin chaperone activity after mild heating in LECs. The α-crystallin isolated from LECs that were not subjected to heating can prevent β- and γ-crystallin from heat-induced amorphous-aggregation. However, following mild heating, the α-crystallin extracted from human LECs showed a relatively lower protective effect against β- and γ-crystallin amorphous-aggregation.

## Discussion

In the experiments reported here, LECs subjected to mild heating could provide an alternative model for studying lens aging, through inducing a diminished regulatory function for α-crystallin in maintaining the lens membrane head group environment, in addition to attenuating its function as a molecular chaperone that exhibits a protective effect on other proteins.

Based on previous studies^6^,^10^,^12^ that have demonstrated that the exposure of intact lenses to mild thermal stress (50 °C) results in changes to the soluble protein content within lens cells, we have developed a lens cell heating method that can model cellular aging.

The increase in temperature during the mild heating of LECs diminishes the ability of α-crystallin to modulate the integrity of cell membranes. With increasing heating times, the membrane head group order of LECs showed increased fluidity. Our data is consistent with that of a study by Zhu *et al*. who showed that similar changes in the membrane head group environment occurred in the human lens with aging^6^,^10^. Therefore, the findings from our study, which indicate that age-related changes in the lens can be replicated by thermal stress, supports our proposal for a mild heating model for lens aging using LECs. Cataract is a pathological opacity caused either by disturbances to the cytoplasm-membrane architecture of lens or by perturbations to the interior region of lens fiber cells of the local short-range order of crystallins^1^. On the other hand, the spatial fluctuations in protein density that lead to lens opacification can be attributed to the osmotic pressure effects on LECs^13^. Previous studies have also suggested that the synthesis of functional α-crystallin has a pronounced stabilizing effect on model membranes^14^, and this evidence supports the speculation that changes in α-crystallin structure and function may affect the normal structure of the LEC membrane, resulting in increased permeability and subsequent water penetration. With the utilization of our cellular heating model, we were able to obtain a more intuitive method for analyzing the changes in cellular membrane head group order during lens aging *in vitro*, and the decreased activity α-crystallin that is necessary for maintaining the integrity of LEC membranes.

Moreover, our study showed that changes in the intracellular distribution and decreased expression of α-crystallin occurred with mild heating in LECs. With increasing duration of heating, α-crystallin gradually accumulated in the cytoplasm and finally around the nucleus. This might represent possible age-related changes that occur in different regions of the lens. Previous studies showed that the outer lens regions are largely unaffected by mild heating, compared to the inner region. Therefore, we speculated that LECs with shorter heating times reacted in a similar way to cells of the outer lens region, while those with longer heating times more closely resembled the inner lens region. The cellular heating model is therefore a useful platform for observing the distribution of crystallins in the lens, as it can be used to simulate responses in cells from different lens regions. The denaturation of α-crystallin was also detected during the mild heating of LECs in our study. These observations are in accordance with previous findings that decreased expression of α-crystallin is implicated in age-related cataract lenses^15^,^16^.

In addition to the denaturation of α-crystallin, thermal stress could reduce the chaperone activity of α-crystallin; sHSPs can protect from stress by binding to some partially unfolded substrates with the ability to integrate with HMW monodisperse or polydisperse oligomers. It is known that the chaperone activity of α-crystallin *in vitro* was primarily detected by Horwitz J *et al*.^5^,^17^. Based on these results, α-crystallin showed the ability to prevent further precipitation and lens opacity by binding to the primarily denatured β-crystallin or γ-crystallin. On evaluation of our results, which indicate that the α-crystallin from heated LECs has a relatively lower protective effect on β- and γ-crystallin after being subjected to heating, our study provides support for the use of a cellular heating model in lens aging studies. Compared to lens tissue models, an aging model based on LECs is more accurate and convenient for large-sample analysis of the chaperone activity changes that occur during lens aging.

We acknowledge that our study has some limitations. We did not discuss the loss in cell viability, which may influence our results through other mechanisms influencing sHSP expression that perhaps did not mimic normal cell aging.

In conclusion, we have demonstrated cellular changes that occur in LECs through a series of cytological and molecular experiments to assess and prove the effectiveness of a cellular model for the study of lens aging, including those changes in membrane head group order in each cell, the real-time observation of crystallin distribution, and the monitoring of functional changes in the chaperone activity of crystallins as a result of aging. According to our study, LECs under mild heating conditions could be considered as an alternative lens-aging model *in vitro*, which can be used to broaden the research field in studying age-related changes in the lens,

## Matetials and Methods

### Cell cultures

The human lens epithelial cells (LECs, SRA01/04) used in this study were purchased directly from the Cell Culture Center of the Shanghai Institutes for Biological Sciences (Chinese Academy of Sciences, Shanghai, China). Briefly, LECs (SRA01/04) were cultured in sterilized 6-well plates with modified high glucose Dulbecco’s Modified Eagle Medium (DMEM, Hyclone) supplemented with 1% Penicillin-Streptomycin (Hyclone) and 15% heat-inactivated fetal bovine serum (Hyclone). Cells were maintained at 37 °C in a humidified atmosphere of 5% CO_2_ and 95% air until semiconfluent monolayers were obtained.

### Cell heating model construction

Based on previous studies on the use of lens tissue heating models to simulate the natural aging process, we further established the cell heating model as follows: lens epithelial cells (SRA01/04) were incubated at 50 °C for 0 min, 15 min, 30 min, 45 min, 60 min, and 75 min. Cells were then analyzed in subsequent experiments, including determination of cell viability, analysis of membrane head group order, and immunofluorescence staining. We also performed the intermittent mild heating part (see supplementary data). In this additional experiment, the heating process was intermittent over time and we observed the cells over longer periods after repeat-periodic short exposure to heat.

### Cell viability analysis

To determine a suitable mild heating time for LECs, the time of incubation at 50 °C was first optimized using the Cell Counting Kit-8 (CCK-8; Dojindo Laboratories, Japan) assay and trypan blue exclusion cell quantitation. The LECs were cultured in 96-well plates and treated at different heating times ranging from 0 min, 15 min, 30 min, 45 min, 60 min, and 75 min. Then, 10 μl of CCK-8 solution was added to each well, and the LECs were maintained at 37°C for 2 h. The absorbance of samples in each well was measured at 450 nm.

### Laurdan labeling of membrane head group order in lens epithelial cells

Laurdan staining and two-photon confocal microscopy was applied for the analysis of the lens membrane head group order in each group to compare the effects of different heating times. Cells incubated at 50 °C were seeded on sterilized coverslips for the indicated time periods (0 min, 15 min, 30 min, 45 min, 60 min, and 75 min). The medium was removed and each dish fitted with a coverslip before incubation with 2 μL Laurdan stock solution in serum-free DMEM (1:1,000 dilution, 5 μM final concentration) at 37 °C in a humidified 5% CO2 atmosphere for 30 min. Coverslips were washed with PBS after draining the medium, then incubated in 4% (wt/vol) paraformaldehyde solution (40 μL) for 15 min at 37 °C in a humidified 5% CO2 atmosphere. The coverslips were then mounted in 20-μL medium (Mowiol; Calbiochem, Germany) on sterilized slides before stored at room temperature overnight in the dark. Before the following analysis, the coverslips were stored at 4 °C.

Images (775 × 775 μm^2^, 1024 × 1024 pixels) were obtained using a TCS SP5 confocal microscope (Leica, Germany) in the following emission ranges: 400–460 nm and 470–530 nm, without margin overlap in the middle and four quadrant (up, down, left and right) regions.

As described in our previous study^6^, generalized polarization (GP) was produced by pairs of Laurdan intensity images using ImageJ software using the formula:


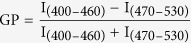


where I = signal intensity. Final GP images were pseudocolored using a graphic editing program (Photoshop; Adobe, Mountain View, CA).

### Immunofluorescence staining of α-crystallin in lens epithelial cells

Immunofluorescence staining was used to detect the expression and localization of αA-crystallin (CRYAA) and αB-crystallin (CRYAB) in human LECs. Cells incubated at 50 °C were seeded on sterilized coverslips for the indicated time periods (0 min, 15 min, 30 min, 45 min, 60 min, and 75 min) and then fixed in 4% paraformaldehyde for 15 min. Cells were incubated with 3% bovine serum albumin in PBS solution for 60 min to block nonspecific interactions. Coverslips were added and followed by addition of mouse anti-human CRYAA monoclonal antibody (ab139337, Abcam), or mouse anti-human CRYAB monoclonal antibody (ab13496, Abcam) and incubated overnight at 4 °C. After washing with PBS, cells were incubated with fluorescent dye-labeled goat anti-mouse IgG secondary antibodies (ab6785, Abcam) at room temperature for 1 h. Anti-fade DAPI solution was added and images were obtained using a fluorescence microscope.

### SDS-PAGE analysis of water-soluble and water-insoluble protein fractions from lens epithelial cells

All procedures were performed at 4 °C unless indicated otherwise. Protein extraction from human lens epithelial cells was performed using RIPA Lysis Buffer (P0013C, Beyotime, Wuhan, China). After centrifugation at 10,000 × *g* for 5 min, the supernatant was recovered and the pellet centrifuged twice as described above. The supernatants recovered after each centrifugation were combined and designated as the WS protein fraction, and the pellet was designated as the WI protein fraction. After determining the protein concentration using the Enhanced BCA Protein Assay Kit (P0010S, Beyotime, Wuhan, China), WS proteins from groups with different heating times were added to 5X SDS-PAGE loading buffer and denatured at 100 °C for 5 min. Then samples were separated by 12% gradient acrylamide sodium dodecyl sulphate-polyacrylamide gel electrophoresis. Protein bands were subjected to Coomassie Blue Fast Staining Solution (P0017, Beyotime, Wuhan, China) for 1 h at room temperature.

### Molecular chaperone function analysis

The molecular chaperone activity of α-crystallin was detected as the percentage difference in relative absorbency (or turbidity) between α-crystallin and its target protein (β-crystallin or γ-crystallin). In each experiment, 200 μg/mL of crystallin in 60 mM sodium phosphate (pH 7.0) was used in a total volume of 500 μl. The relative light scattering was measured at 360 nm. Measurements were made using a Nanodrop with an ultra-sensitive tip. Two groups of α-crystallin samples were separately dissolved in buffer solution (0.05 mol/L NaH_2_PO_4_, 0.2 mol/L KCl, 2 mmol/L EDTA, pH 6.7) with either β-crystallin or γ-crystallin in Eppendorf tubes according to the molecular weight ratios (1:1). During mild heating at 50 °C, the total volume was 500 μL, and scattering was recorded at 5-min intervals until heating for 75 min. Blank controls were used for each group using lysozyme as the control protein.

### Statistical analysis

Data was presented as mean values ± standard deviations (SD). Statistical analyses were performed using SPSS version 20.0 (SPSS Inc., Chicago, IL, USA). A *P* value of <0.05 was considered statistically significant in all cases.

## Additional Information

**How to cite this article**: Zhang, K. *et al*. Effect of Mild Heating on Human Lens Epithelial Cells: A Possible Model of Lens Aging. *Sci. Rep.* **6**, 33917; doi: 10.1038/srep33917 (2016).

## Supplementary Material

Supplementary Information
