# Supplementary material for: Effect of Mild Heating on Human Lens Epithelial Cells: A Possible Model of Lens Aging
Source: Sci Rep. 2016 Oct 11;6:33917. doi: 10.1038/srep33917 (PMC5057073; doi:10.1038/srep33917)
Supplement: Supplementary Information [file srep33917-s1.pdf]

Effect of Mild Heating on Human Lens Epithelial Cells: A Possible Model of Lens Aging

Authors: Keke Zhang† [1,2], Xiangjia Zhu† [1,2], Yi Lu* [1,2]

† These authors contributed equally to this work.

* Corresponding author.

[1]Department of Ophthalmology, Eye and ENT Hospital, Fudan University, Shanghai, China

[2]Key Laboratory of Myopia, Ministry of Health PR China, Shanghai, China; and Shanghai Key Laboratory of Visual Impairment and Restoration, Fudan University, Shanghai, China.

Short title: Mild Heating on Human Lens Epithelial Cells

Address for correspondence and reprint requests: E-mail: luyieent@126.com (YL)

Key words: lens epithelial cell; mild heating; cataract; crystallin

Intermittent mild heating process.

In this new part, the heating process was intermittent over time and we observed the cells over longer periods after repeat-periodic short exposure to heat. Firstly, we added the results of three groups of short exposure time to heat in the revised manuscript, including 2min, 5 min, 10 min as the heating time. Secondly, we also intermittent the heating process for 15 min at 37°C between every 5-minute-heating at 50°C, including the following 4 intermittent heating groups, 5 min intermittent mild heating for 2 times, 4 times, 6 times and 8 times. The control group was the LECs cultured at 37°C.

With the new method, we found reduced early loss of cell viability but similar finding when cells were heated for 60 min or longer. In supplementary data, we also provided the additional results of Laurdan labeling (Supplementary Figure 1 and 2) and SDS-PAGE (Supplementary Figure 3 and 4) of LECs under intermittent mild heating. The results of short period heating for 2min, 5min and 10 min (Supplementary Figure 3) showed a similar tendency as Fig.5. The overall tendency of membrane fluidity changes during intermittent heating were similar to continuous mild heating. However, we found that intermittent heating groups showed a relatively lower expression in the 70-kDa band compared to the continuous groups of same heating time. For example, LECs heated for 5min for 2 times, showed a relatively lower expression in the 70-kDa band when compared to those heated for 10 min.

Figure Legends.

Supplementary Figure 1. The effect of short-period mild heating on membrane GP values of lens epithelial cells. Representative pseudocolored GP images of lens epithelial cells indicate changes in membrane head group order following intermittent mild heating.

Supplementary Figure 2. The effect of intermittent mild heating on membrane GP values of lens epithelial cells. Representative pseudocolored GP images of lens epithelial cells indicate changes in membrane head group order following intermittent mild heating.

Supplementary Figure 3. Changes in water-soluble protein expression as a result of short-period mild heating of increasing duration.

Supplementary Figure 4. Changes in water-soluble protein expression as a result of intermittent mild heating of increasing duration.

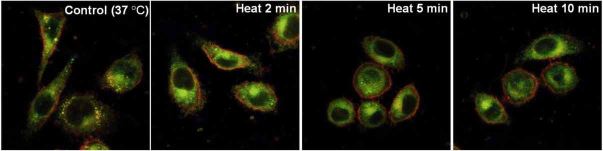

Supplementary Figure 1

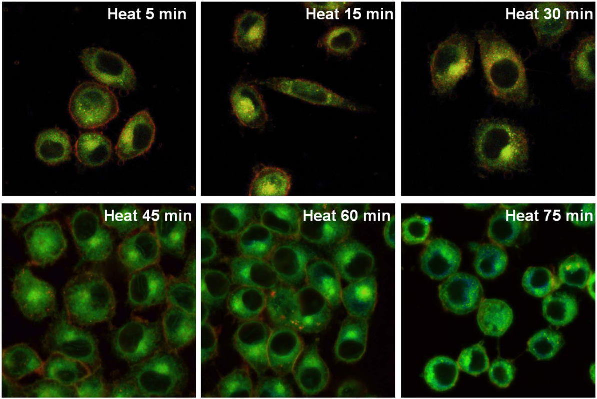

Supplementary Figure 2

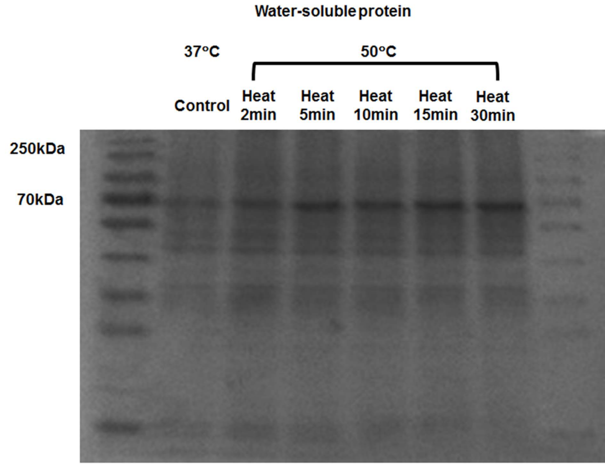

Supplementary Figure 3

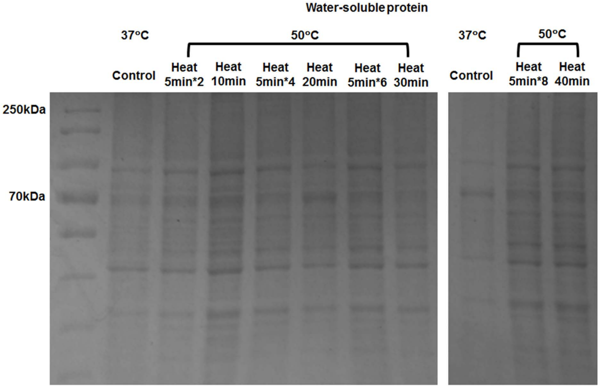

Supplementary Figure 4
